# Supplementary material for: The Arrestin-like Protein palF Contributes to Growth, Sporulation, Spore Germination, Osmolarity, and Pathogenicity of Coniella vitis
Source: J Fungi (Basel). 2024 Jul 22;10(7):508. doi: 10.3390/jof10070508 (PMC11277687; doi:10.3390/jof10070508)
Supplement: Supplementary file 1 [file jof-10-00508-s001.zip › Table S1.pdf]

Table S1 List of primers used qPCR in this study

Gene ID	Gene name	Forward	Reverse
CV_V002577	Metal-independent alpha-mannosidase (GH125)	GGTTCTCGGAGCAGTCAATAC	GACCGTTGTAAGAGACCTTGAG
CV_V004338	palC	CGACGCCCTCAAGAAATACAA	CAAGGAGGTATGGGAGGTAAGA
CV_V007812	pmeB	CTTCTACTCGTGCGGTTTCTAC	GTACAGGGTACCAAAGCCATAC
CV_V003186	pmeA	TACGCAGACTCTGGCTACTA	CTCGATGTAAGACTGGGCATAG
CV_V004126	pectin lyase-like protein	CGGCCAAGATATCTCCTCAATC	GGTGGTTGCAGATCCAGTT
CV_V008718	beta-glucosidase	GATTTCCGCGTGCAGTATTTC	TCGGCCCACTCAAAGTTATC
CV_V009031	exgD	CGAAGCACGGCACAATAAAC	GGATAGGGTCTGACGAGAAGA
CV_V004839	Cellulase (glycosyl hydrolase family 5)	TGAGGTCGTCGTTGTTCTTATC	TGTGCCATAGACGCCAAAT
CV_V001325	pacC	CTGTCCACACTGGAGGTATTG	GCATTGGTGGTAGGTAGTAGTG
CV_V008724	palB	CCTCAACTGGAACCCTACATTAT	AAGGAGAGGTGAAGCTGTATTG
CV_V003488	palF	GCCATTAGCGTCTGGTTCTT	GTGCCAAAGGACGCAGTAT
CV_V008641	palH	GAAGCTACGGGACCGTATAAAG	CTGCACGTAGCTGTCGTAAT
CV_V004811	palA	CCTGGGTGATCATACGGATTT	CTAGGCGATCTCGTCTTTCTTC
CV_V007795	palI	TTCGGTGTCTTTGGCTACTG	GTGGACTTGGGAAGGTCAAA
