# Supplementary material for: The Arrestin-like Protein palF Contributes to Growth, Sporulation, Spore Germination, Osmolarity, and Pathogenicity of Coniella vitis
Source: J Fungi (Basel). 2024 Jul 22;10(7):508. doi: 10.3390/jof10070508 (PMC11277687; doi:10.3390/jof10070508)
Supplement: Supplementary file 1 [file jof-10-00508-s001.zip › text S1.pdf]

**1. Protein sequence of PalF in *C.vitis* GP1**

MVLHSSDNDPAAALDDQPPSRSFFSRFALPIRSRNVRNVIDFHIRPAEPHRKYNAGDAVRG

AVILTVVKPIRVTHLTVSLRGSVRVYKNSNAANEPASNADIASDGISRFRFLGNGYASLFQD

EQVLCAEGKIDTGKYEFEFELMFPEGGLPSSIEFERGAIAYNITAILTRPSPTSPTISCERKIDL

VEQIDVGHLAPPPQRTIYLEPISKKSRRKKQPASSISERQNPASDANTSEVAYDGDSTRAAD

TSTDGGSNPLEESSQDAQQNPRSPVVSDLRSEVSGESVVSSSSRGADLNGQAGSVGTLQT

SGGRRTISGDKTIVTTIELMKGGCLAGDALPIRVNIQHTKPIRSMHGIIVTLYRQGRVDSAPP

LSLFGNLSKEELRRLEKDEYLPKSKTGLGGLSLTSAGSCSVFRKDLSQSFTPLIIDPGTFTTS

ITTSVRVPEHAFPSIRGVPGEMISFKYRLEVIVDLGGKVSNLIQIGQNARLAAGVGGANLSS

SRDLAAGVTSFDGSLINTDTIKREKGVIFDSFEVIVGTKDSKRRGKAAMAPAQSVRTEYHD

TTDYNESGQWPTGPEYDGYGGEDSYHYHHVDVPAQPGTHYPYWHGGPREPSQQASAPH

YIPPPQFPDESALSEKERIRMAEQRLLPSQPQHTHEPTAGPSQSTNDEGAGPSIAAATLPVQ

ESLISSRPTAPSMNDLSADSSVPEATSSDDKQELERQRLLTEASAPPEFPNDYDAGVGSSSG

HMAGPSAPSAPPPMAVHEEIAEPSAPALSEEDEYGQQYAYNDVAAAHGSTQASAPPLEPLP

AYQR

**2. Gene sequence of palF in *C.vitis* GP1**

ATGGTCCTTCATTCTAGCGACAATGACCCTGCCGCCGCGCTCGATGACCAGCCGCCATC
GCGATCCTTTTTCTCGCGCTTCGCTCTCCCTATCCGCTCGCGAAACGTGCGAAATGTCA
TCGACTTCCACATTCGCCCCGCCGAACCGCATCGCAAATACAATGCAGGCGACGCTGT
GAGGGGCGCCGTTATTCTGACCGTCGTCAAGCCCATTCGGGTCACACACCTCACGGTC
AGCTTGCGCGGCTCGGTGCGCGTTTACAAGAACTCGAATGCCGCCAACGAGCCCGCTA
GCAATGCCGACATTGCCTCGGATGGCATTTCTCGCTTCAGATTCCTCGGCAACGGCTAT
GCTTCCTTGTTTCAAGACGAACAAGTCCTCTGCGCCGAAGGCAAAATAGACACCGGC
AAATATGAGTTTGAGTTCGAGTTGATGTTCCCAGAGGGAGGGCTGCCTAGTAGCATCG
AGTTCGAACGAGGGGCAATAGCTTACAATATCACTGCGATTCTCACAAGACCATCGCC
AACCTCACCCACAATCAGCTGCGAACGCAAAATCGATCTGGTGGAGCAAATCGATGTC
GGCCACCTTGCGCCGCCTCCACAACGGACAATATACCTTGAGCCCATATCCAAAAAATC
GAGAAGGAAGAAGCAGCCCGCCTCGTCCATCTCTGAAAGACAAAACCCGGCATCTGA
CGCAAACACAAGCGAAGTTGCTTATGACGGCGATTCAACACGAGCTGCAGACACGTC
GACAGATGGTGGTTCGAATCCGCTCGAAGAATCCAGCCAGGATGCGCAACAAAATCCT
AGAAGCCCGGTAGTCAGCGACTTGAGGAGCGAAGTCAGTGGAGAAAGCGTTGTGAG
CAGCAGCTCAAGAGGAGCGGATTTGAACGGGCAGGCTGGGAGTGTGGGGACTCTCCA
GACGAGCGGCGGCAGAAGAACAATTTCTGGGGACAAGACGATTGTCACGACGATCGA
GCTGATGAAAGGTGGCTGCTTGGCTGGCGATGCGTTACCTATCAGGGTGAACATTCAG
CACACGAAACCCATACGGAGCATGCACGGCATTATTGTCACCTTGTACCGGCAGGGCC

GAGTCGACTCGGCTCCACCGTTATCACTGTTTGGGAACCTGTCGAAAGAGGAGCTGCG
CAGATTGGAGAAGGATGAGTATTTGCCAAAGTCCAAGACGGGCCTAGGTGGTCTGTCA
TTGACATCAGCCGGGTCGTGCAGCGTCTTTCGAAAGGATCTTTCTCAGTCGTTCACGC
CGCTGATCATCGACCCGGGGACTTTTACTACGAGCATCACAACATCAGTACGAGTACCA
GAGCACGCCTTCCCTTCAATCAGAGGGGTTCCGGGAGAAATGATTAGTTTCAAATACC
GTTTGGAGGTTATTGTGGACCTGGGTGGTAAGGTGTCGAATTTGATACAAATCGGACA
GAATGCAAGACTCGCCGCCGGTGTTGGTGGCGCCAACTTATCCTCGTCAAGAGACTTG
GCAGCCGGTGTCACCTCGTTTGATGGTAGCTTGATCAACACAGACACAATCAAACGGG
AAAAGGGCGTCATCTTTGACTCATTTGAGGTCATTGTGGGCACAAAAGACAGCAAAA
GACGGGGCAAGGCAGCAATGGCGCCTGCACAATCGGTGCGTACAGAGTATCACGACA
CCACCGACTATAATGAATCTGGCCAGTGGCCGACAGGGCCCGAGTATGATGGTTATGGC
GGCGAGGATTCCTATCATTACCACCATGTGGATGTGCCAGCCCAGCCCGGCACGCACTA
CCCCTACTGGCACGGTGGTCCCAGAGAACCATCTCAGCAGGCATCTGCACCTCACTAC
ATACCTCCTCCGCAGTTCCCAGACGAGAGTGCTCTATCTGAAAAGGAACGAATCCGCA
TGGCAGAACAGAGACTTCTTCCTAGCCAACCACAACACACTCACGAACCCACTGCTG
GTCCTTCTCAATCCACAAACGACGAAGGTGCTGGTCCATCTATTGCAGCTGCAACACT
GCCAGTGCAAGAATCCCTTATTTCATCTAGGCCAACAGCACCGAGTATGAACGATTTGA
GCGCAGACTCAAGTGTACCCGAGGCCACCTCGTCAGACGACAAGCAGGAGCTGGAGA
GGCAGAGACTGTTGACTGAAGCAAGTGCACCGCCCGAGTTTCCCAATGACTACGATGC
AGGGGTGGGAAGCAGCAGCGGGCACATGGCAGGACCAAGCGCACCAAGCGCGCCGC
CGCCAATGGCGGTCCACGAAGAGATCGCCGAGCCATCTGCGCCGGCCCTCAGTGAAG
AGGACGAATATGGTCAGCAGTATGCATACAATGATGTTGCTGCTGCCCATGGATCCACA
CAGGCATCCGCTCCACCGCTAGAGCCATTACCTGCCTATCAGCGGTGA
