# Supplementary figures and images for: The Arrestin-like Protein palF Contributes to Growth, Sporulation, Spore Germination, Osmolarity, and Pathogenicity of Coniella vitis
Source: J Fungi (Basel). 2024 Jul 22;10(7):508. doi: 10.3390/jof10070508 (PMC11277687; doi:10.3390/jof10070508)

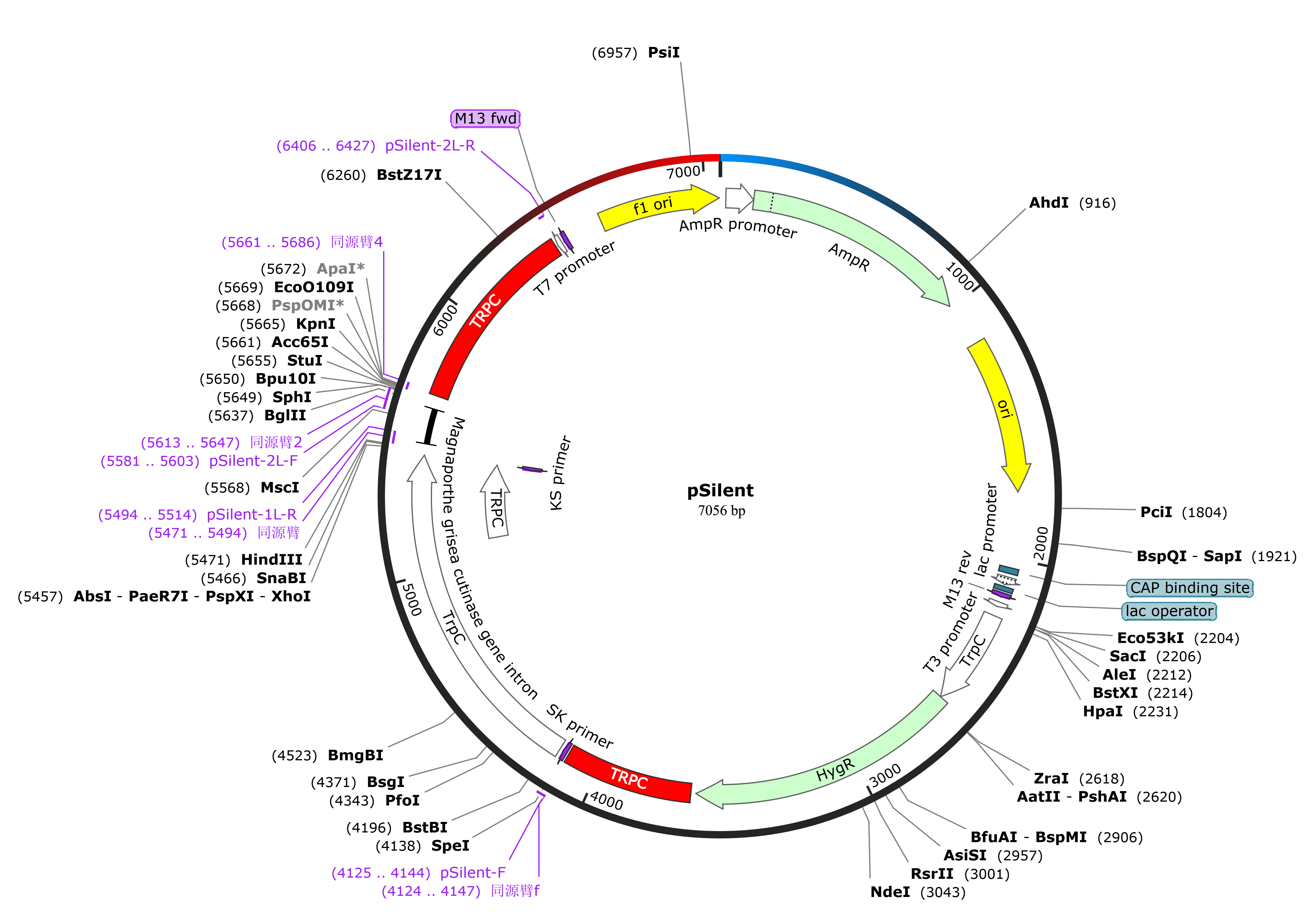

Supplement: Supplementary file 1 [file jof-10-00508-s001.zip › Figure S1.tif]

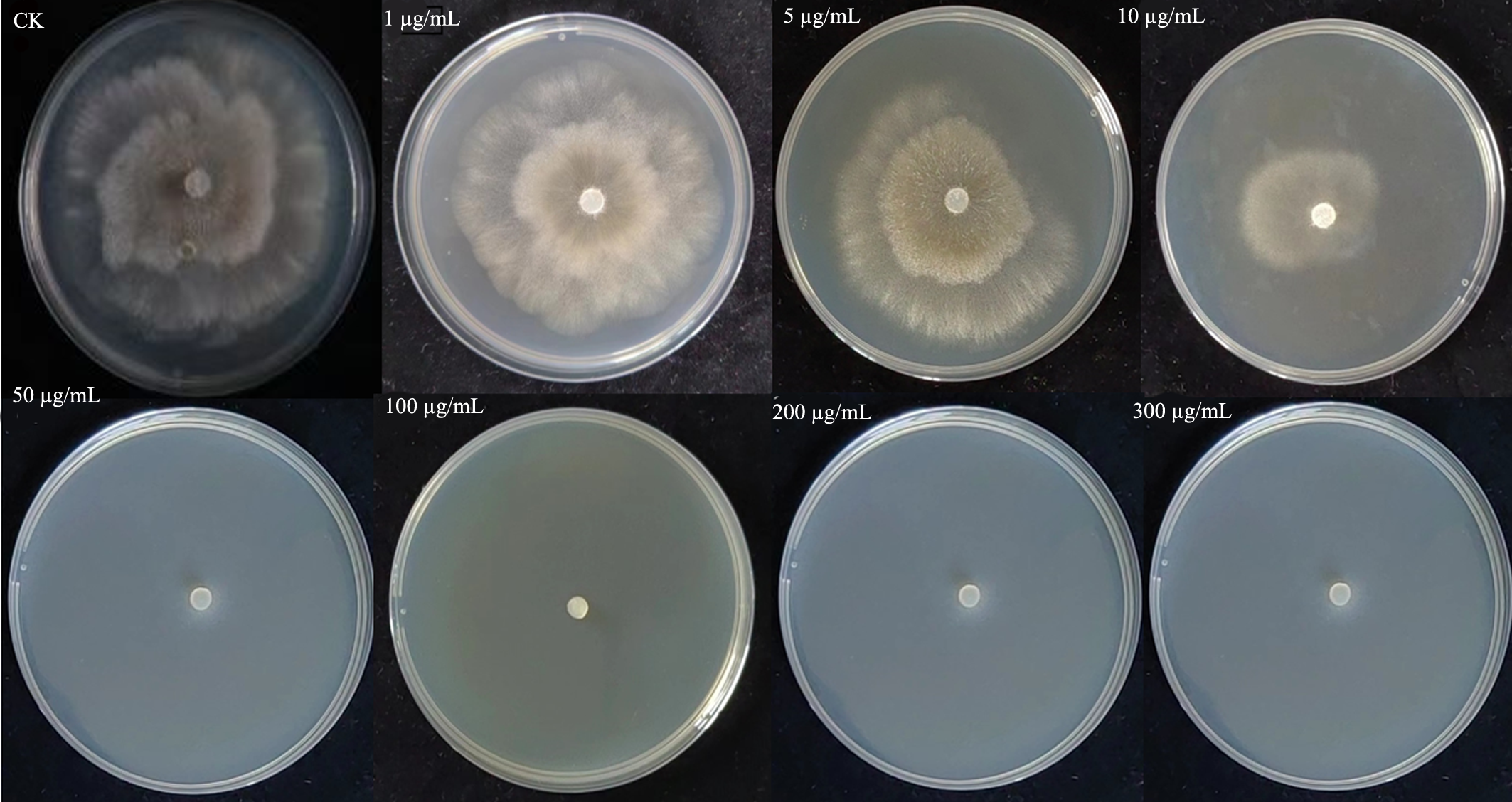

Supplement: Supplementary file 1 [file jof-10-00508-s001.zip › Figure S2.tif]

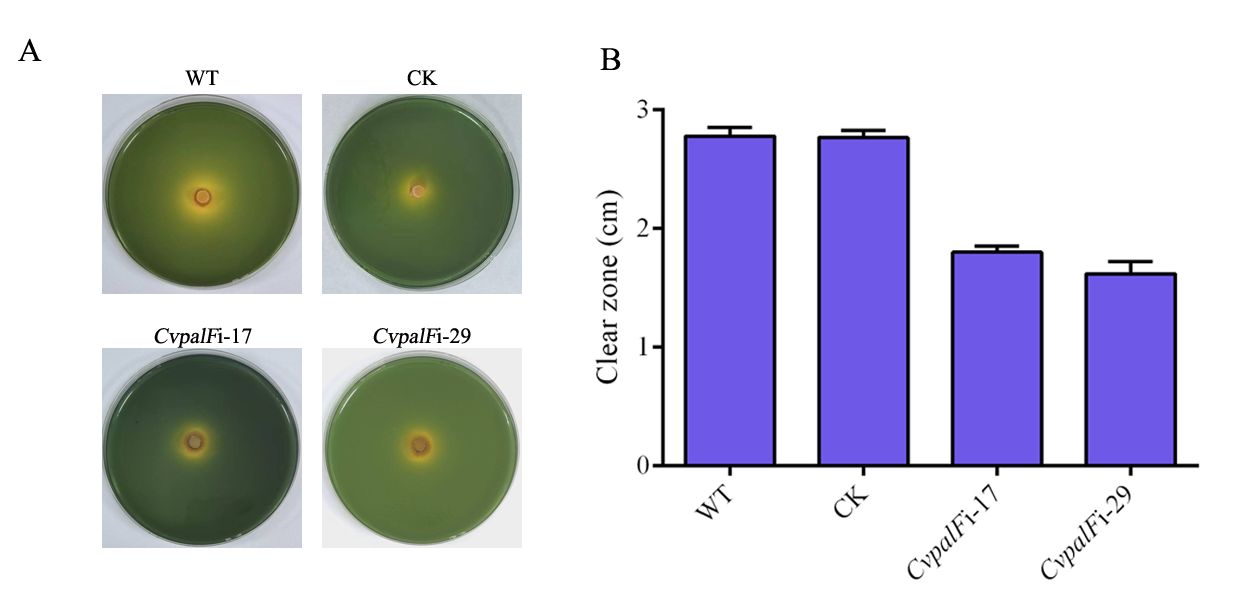

Supplement: Supplementary file 1 [file jof-10-00508-s001.zip › Figure S3.tif]

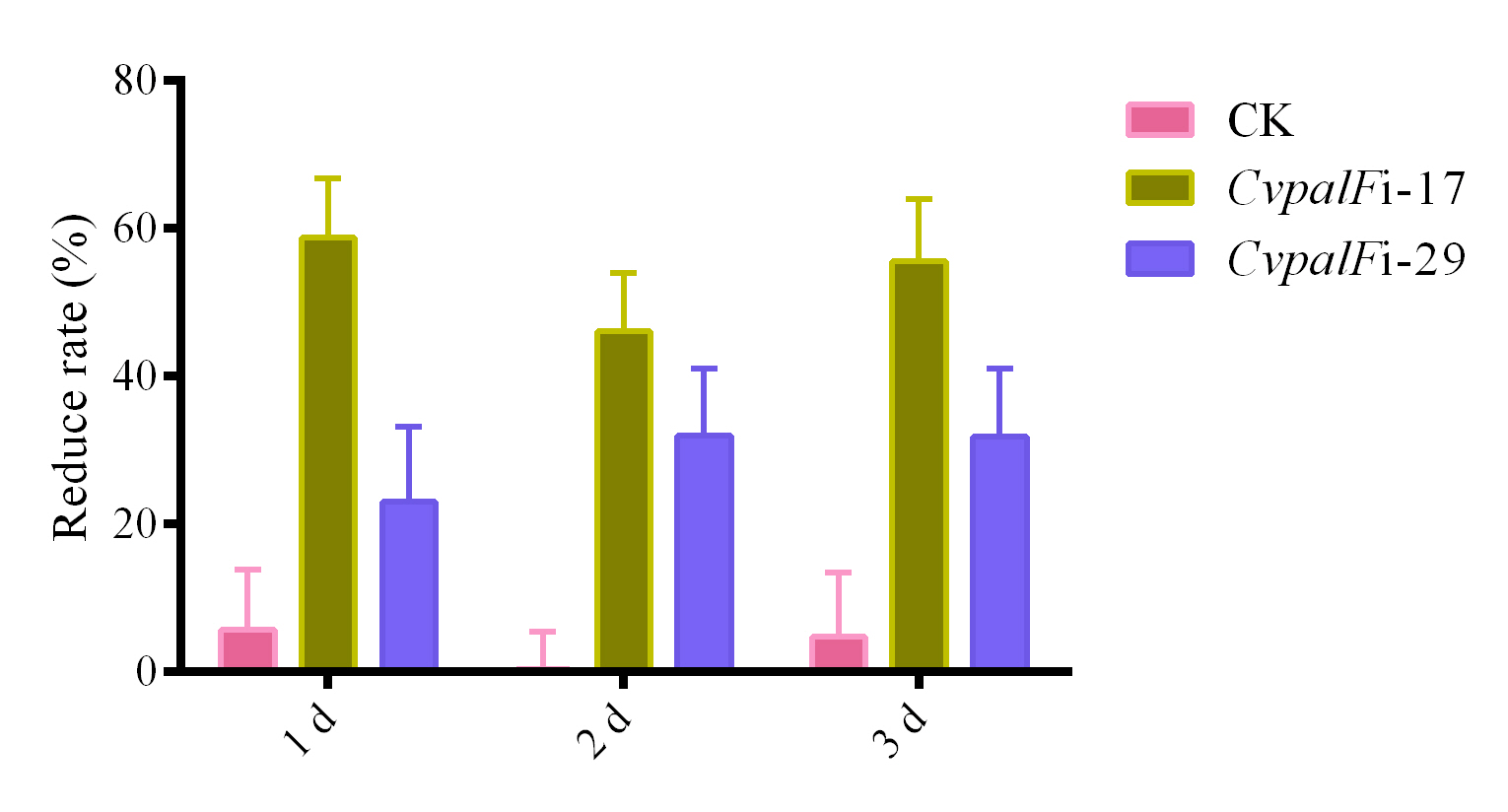

Supplement: Supplementary file 1 [file jof-10-00508-s001.zip › Figure S4.jpg]

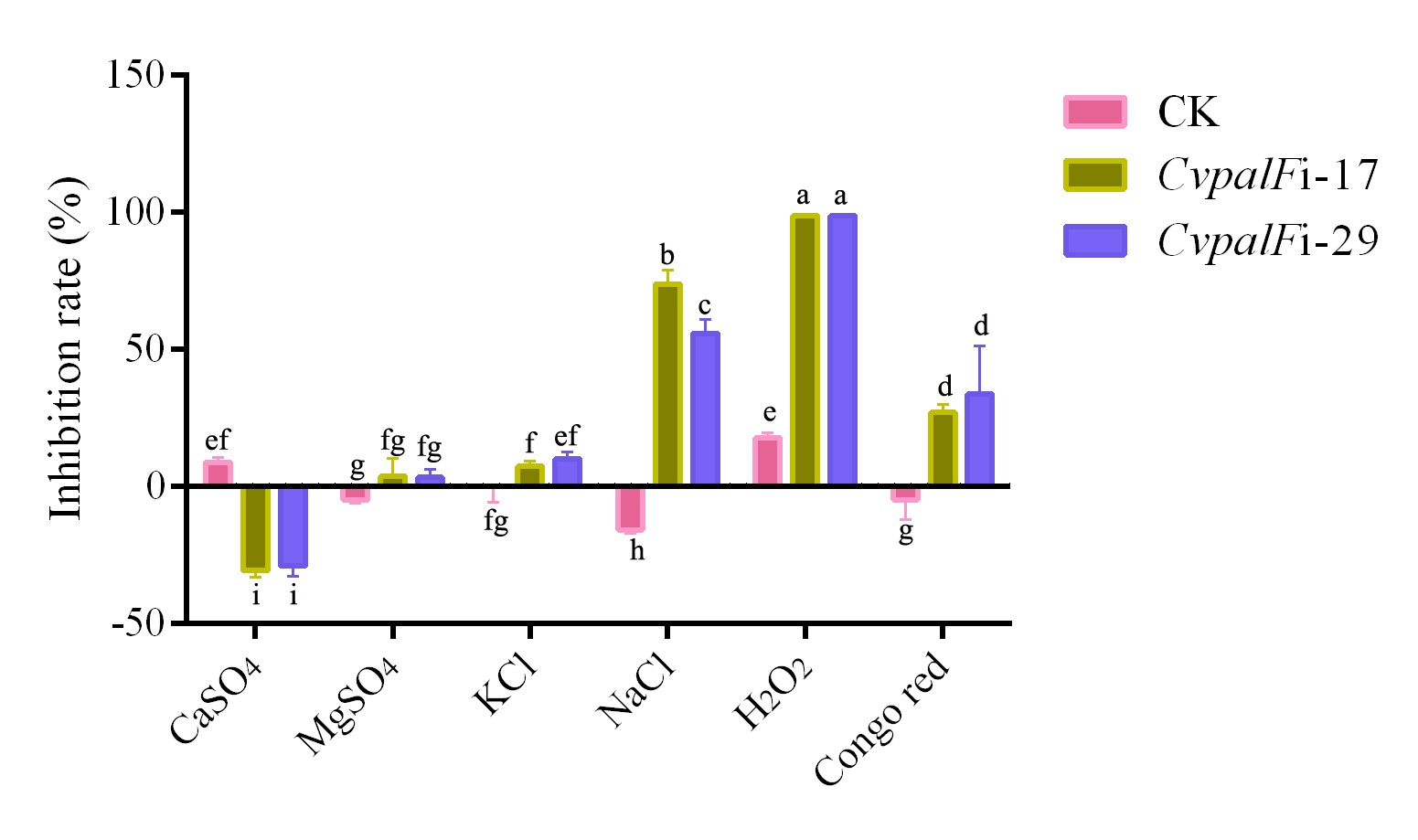

Supplement: Supplementary file 1 [file jof-10-00508-s001.zip › Figure S5.tif]
